# Green Visual Sensor of Plant: An Energy-Efficient Compressive Video Sensing in the Internet of Things

**DOI:** 10.3389/fpls.2022.849606

**Published:** 2022-02-28

**Authors:** Ran Li, Yihao Yang, Fengyuan Sun

**Affiliations:** ^1^School of Computer and Information Technology, Xinyang Normal University, Xinyang, China; ^2^Guangxi Key Laboratory of Wireless Wideband Communication and Signal Processing, Guilin University of Electronic Technology, Guilin, China

**Keywords:** Internet of Things, visual sensor, Compressive Video Sensing, context extraction, linear recovery, plant monitoring

## Abstract

Internet of Things (IoT) realizes the real-time video monitoring of plant propagation or growth in the wild. However, the monitoring time is seriously limited by the battery capacity of the visual sensor, which poses a challenge to the long-working plant monitoring. Video coding is the most consuming component in a visual sensor, it is important to design an energy-efficient video codec in order to extend the time of monitoring plants. This article presents an energy-efficient Compressive Video Sensing (CVS) system to make the visual sensor green. We fuse a context-based allocation into CVS to improve the reconstruction quality with fewer computations. Especially, considering the practicality of CVS, we extract the contexts of video frames from compressive measurements but not from original pixels. Adapting to these contexts, more measurements are allocated to capture the complex structures but fewer to the simple structures. This adaptive allocation enables the low-complexity recovery algorithm to produce high-quality reconstructed video sequences. Experimental results show that by deploying the proposed context-based CVS system on the visual sensor, the rate-distortion performance is significantly improved when comparing it with some state-of-the-art methods, and the computational complexity is also reduced, resulting in a low energy consumption.

## 1. Introduction

In the Internet of Things (IoT), the plant propagation process or plant growth can be monitored by visual sensors. One benefit from the framework of IoT, a large amount of data on the plant can be gathered in a central server, and the valuable information can be achieved by analyzing the data in real-time. However, with the limited processing capabilities and power/energy budget of visual sensors, it is a challenge for video monitoring of plant to compress large-scale video sequences by using the traditional codec, e.g., H.264/AVC and HEVC (Sullivan et al., 2012), so the existing works have developed low-complexity and energy-efficient video codecs, in which Distributed Video Coding (DVC) (Girod et al., 2005) and Compressive Video Sensing (CVS) (Baraniuk et al., 2017) have attracted more attention in industry and academia. Different from DVC, CVS dispenses with the feedback and virtual channels (Unde and Pattathil, 2020), which makes the codec framework simpler. Meanwhile, CVS provides a low-complexity encoder because of its theoretic foundation, Compressive Sensing (CS) (Baraniuk, 2007), realizes the capture of video frames at a rate significantly below the Nyquist rate. Currently, many researchers recognize that CVS is a potential scheme to compress the video sequences in the IoT framework, and especially for wireless video monitoring of plants, the CVS scheme can assist visual sensors to efficiently reduce the energy consumptions, however, its rate-distortion performances are still far from satisfactory.

The objective of this article is to improve the rate-distortion performance of CVS, providing high-quality video monitoring of plants with low energy consumption. To achieve this objective, the existing works focus on how to design excellent recovery algorithms, and they are keen on mixing various advanced tools into the CVS framework, e.g., the latest popular Deep Neural Network (DNN) (Palangi et al., 2016; Zhao et al., 2020; Tran et al., 2021). Though effective, they bear a heavy computational burden. Different from these works, we try to exploit the capability of CS to capture important structures, improving the reconstruction quality only armed with some simple recovery algorithms. It is well known that the context feature (Shechtman and Irani, 2007; Romano and Elad, 2016) is a good structure for visual quality, and, therefore, in this article, we focus on how to fuse contexts into CVS for an obvious improvement of reconstruction quality.

Compressive Video Sensing consists of three essential steps including CS measurement, measurements quantization, and reconstruction. CS measurement is a process of randomly sampling each video frame, in which the block-based (Gan, 2007; Bigot et al., 2016) or structurally (Do et al., 2012; Zhang et al., 2015) random matrix is often used to ensure the small memory requirement. Output by CS measurement, all measurements are required to be quantized as bits, then transmitted to the decoder. The straightforward solution to incorporating quantization into CVS is simply to apply Scalar Quantization (SQ), but it brings a big error. For block-based sampling, Differential Pulse Code Modulation (DPCM) (Mun and Fowler, 2012) can be used, and it exploits the correlations between blocks to improve the rate-distortion performance. Based on DPCM, many works also proposed some efficient predictive schemes (Zhang et al., 2013; Gao et al., 2015) to quantize CS measurements. Reconstruction is deployed at the decoder, and it uses quantized measurements to reconstruct the video sequence by the CS recovery algorithm. At present, the reconstruction can be implemented by one of the three types: frame-by-frame (Chen Y. et al., 2020; Trevisi et al., 2020), three-dimensional (3D) (Qiu et al., 2015; Tachella et al., 2020), and distributed strategies (Zhang et al., 2020; Zhen et al., 2020). The frame-by-frame reconstruction performs a CS recovery algorithm to reconstruct each video frame independently, and it has a poor rate-distortion performance due to neglecting the correlations between frames. The 3D reconstruction designs some complex representation models to once reconstruct a whole video sequence or a Group Of Pictures (GOP), e.g., Li et al. (2020) proposed the Scalable Structured CVS (SS-CVS) framework, which learns the union of data-driven subspaces model to reconstruct GOPs. However, it has a defect in 3D reconstruction that the huge memory and high computational complexity are required to be invested at decoder. Derived from the decoding strategy of DVC, the distributed reconstruction divides the input video sequence into non-key frames and key frames and reconstructs each non-key frame by the CS recovery algorithm with the aid of its neighboring key frames. With a small memory and a low computational complexity, the distributed reconstruction improves the rate-distortion performance by exploiting the motions between frames, so many existing works focus on it to design the CVS systems, e.g., Ma et al. proposed the DIStributed video Coding Using Compressed Sampling (DISCUCS) (Prades-Nebot et al., 2009), Gan et al. proposed the DIStributeCOmpressed video Sensing (DISCOS) (Do et al., 2009), Fowler et al. proposed the Multi-Hypothesis Block CS (MH-BCS) system (Chen et al., 2011; Tramel and Fowler, 2011; Azghani et al., 2016), etc. The core of distributed reconstruction is the Multi-Hypothesis (MH) predictive technique, which uses a linear combination of blocks in key frames to interpolate the blocks in non-key frames. As one of the state-of-the-art techniques, the MH prediction is widely applied to distributed reconstruction. Recently, some works try to modify the implementation of MH prediction, e.g., Chen C. et al. (2020) added the iterative Reweighted TIKhonov-regularized scheme into MH prediction (MH-RTIK), causing a significant improvement of CVS performance. CS theory indicates that the precise recovery requires enough CS measurements. With insufficient CS measurements, the excellent CS recovery algorithm still cannot prevent the degradation of reconstruction quality, however, by adaptively allocating CS measurements based on local structures of the image, a simple recovery algorithm can also provide a good reconstruction quality (Yu et al., 2010; Taimori and Marvasti, 2018; Zammit and Wassell, 2020). Judging from the above facts, the adaptive allocation is a potential way to improve the rate-distortion performance of the CVS system with a light codec.

This article presents a context-based CVS system, of which the core is the allocation of CS measurements adapted by context structures at the encoder. Based on these adaptive measurements, by combining linear estimation and MH prediction into distributed reconstruction, the decoder provides a satisfying reconstruction quality with low memory and computational cost. The contributions of the proposed context-based CVS is to solve the following issues:

1. How to extract the context structures from CS measurements? Traditional methods use pixels to compute the context features, but it costs lots of computations at the encoder, resulting in impracticality for CVS. Especially when the encoder is realized by Compressive Imaging (CI) devices (Liu et al., 2019; Deng et al., 2021), due to the unavailability of original pixels, it is impossible to perform the traditional methods. Considering the low dimensionality and availability of CS measurements, it is practical in CVS to extract context structures from CS measurements.
2. How to adaptively allocate CS measurements by context structures? Contexts measure the correlations between pixels, and their distribution reveals some meaningful structures, e.g., smoothness, edges, textures, etc. With the same recovery quality, fewer necessary measurements are required for simple structures and more for complex structures. According to the distribution of contexts, an efficient allocation is designed to avoid insufficiency or redundancy of measurements.
3. How to quantize the adaptive measurements? Adaptive allocation makes blocks have different numbers of CS measurements, as a result, the traditional prediction cannot be applied to quantization. Due to the insufficient capability of SQ, an appropriate prediction scheme is required to reduce the quantization error.

Experimental results show that the proposed context-based CVS system outputs the high-quality reconstructed video sequences when monitoring plant growth or propagation and improves the rate-distortion performances when compared with the state-of-the-art CVS systems, which demonstrates the effectiveness of context-based allocation for CVS.

The rest of this article is organized as follows. Section 2 briefly overviews Plant Monitoring System, CVS, and describes the traditional method to extract context features. Section 3 presents the proposed context based CVS system. Experimental results are provided in Section 4, and we conclude this article in Section 5.

## 2. Related Works

### 2.1. Plant Monitoring System

In modern agriculture, it is essential to monitor plant propagation or growth for guaranteeing productivity. The labor costs can be efficiently reduced by automatically capturing the architectural parameters of the plant, so more and more attention has been paid to the design of the plant monitoring system (Somov et al., 2018; Grimblatt et al., 2021; Rayhana et al., 2021). Early, lots of systems are designed to monitor the various environmental parameters on plant growth, such as humidity, temperature, solar illuminance, etc., e.g., James and Maheshwar (2016) used multiple sensors to measure the soil data of plants and transmitted these data to the mobile phone by Raspberry Pi; Okayasu et al. (2017) developed a self-powered wireless monitoring device that is equipped with some environmental sensors; Guo et al. (2018) added big-data services to analyze the environmental data on plant growth. These environmental parameters indirectly indicate the process of plant growth, and they cannot record the visual scenes on plant growth, resulting in the unavailability of the physical structure parameters on plants. To realize the visual monitoring of plants, some works have started to integrate the visual sensors into the plant monitoring system, e.g., Peng et al. (2022) used the binocular camera to capture video sequences on a plant and used the structure from motion method (Piermattei et al., 2019) to extract the 3-D information of a plant; Sajith et al. (2019) designed a complex network to derive the plant growth parameters from the monitoring images; Akila et al. (2017) extracted the plant color and texture by the visual monitoring system. From the above, it can be seen that the visual sensor or camera is used to capture the video sequences on plant growth, and these video sequences are compressed as bitstream which is transmitted to the IoT cloud for further analyzing. As the core of visual sensors, the video compression is a major energy consumer, so a challenge that we face for the visual monitoring system of the plant is to design an energy-efficient video coding scheme to extend the working time of the visual sensor. In the framework of IoT, CVS is a potential coding scheme to reduce the energy consumption of visual sensors. The following briefly overviews the CVS systems.

### 2.2. CVS System

Compressive Video Sensing is the marriage of CS theory and DVC, which reduces the encoding costs and enhances the robustness to noise, thus becoming a potential video codec for wireless visual sensors. At the encoder, to satisfy low complexity and fast computation, the block-based CS sampling is performed on each video frame independently, i.e., the *i*th video frame ***f***_*i*_ of size *N*_1_ × *N*_2_ is partitioned into non-overlapping blocks of size *B* × *B*, each block is vectorized as ***x***_*i,j*_ of length *N*_*b*_, and the CS measurements ***y***_*i,j*_ of ***x***_*i,j*_ are output by

(1) $$\begin{array}{l} y_{i,j}=\Phi_{i,j}\cdot x_{i,j} \end{array}$$

where ***Φ***_*i,j*_ is called as the measurement matrix and can be constructed by some random matrices, e.g., Gaussian, Bernoulli, structural random matrix, etc. By setting the length of ***y***_*i,j*_ to be *M*_*i,j*_, the size of ***Φ***_*i,j*_ is fixed to be *M*_*i,j*_ × *N*_*b*_, and the subrate *S*_*i*_ of ***f***_*i*_ is defined as

(2) $$\begin{array}{l} S_{i}=\frac{M_{i}}{N}=\frac{\sum_{j=1}^{J}M_{i,j}}{N_{1}\times N_{2}} \end{array}$$

where *N* is the number of total pixels in ***f***_*i*_, *M*_*i*_ is the number of CS measurements for ***f***_*i*_, and *J* is the number of blocks in ***f***_*i*_. In CI application, an optical device is designed to perform Equation (1), and directly output the CS measurements. To ensure a stable recovery, *L* video frames are gathered to form a GOP, in which the first frame, called the key frame, is set to be a high subrate, and others, called the non-key frame, are set to be a low subrate. After quantization, all CS measurements of GOP are packaged and transmitted to decoder.

**Figure 1:**
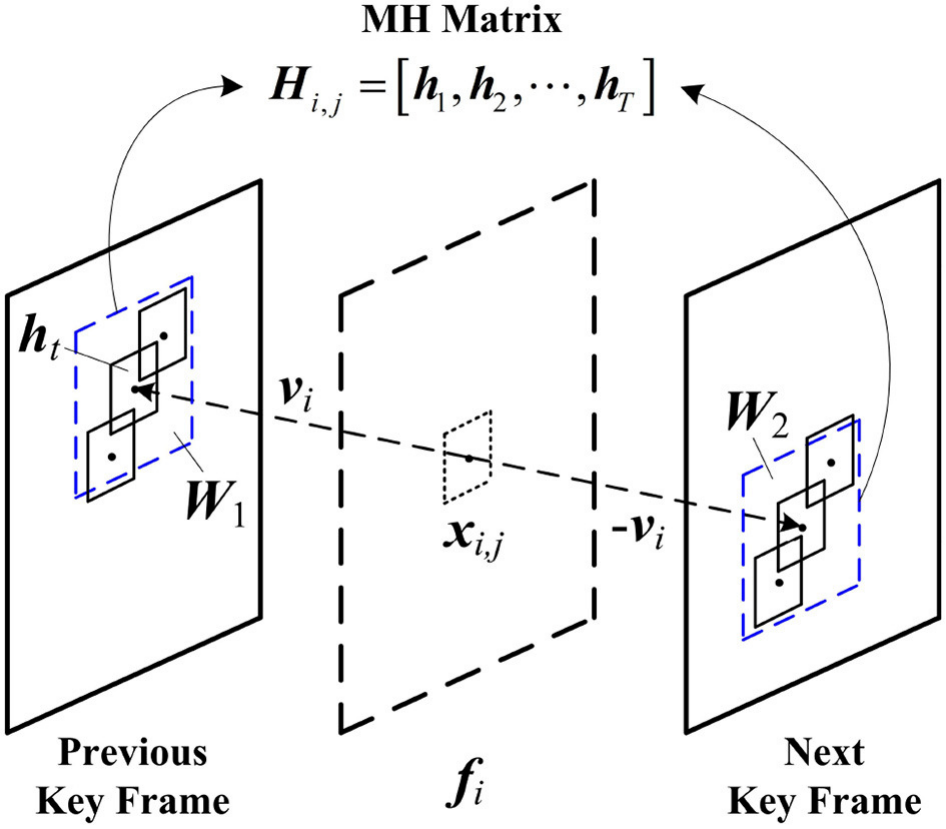
Mechanism of Multi-Hypothesis (MH) prediction.

At the decoder, by using the received CS measurements, the frame-by-frame, 3D, or distributed strategy is performed to reconstruct the GOP. For frame-by-frame, the reconstruction model can be represented by

(3) $$\begin{array}{l} {\left\{{\hat{x}}_{i,j}\right\}}_{j=1}^{J}=arg\underset{{\left\{x_{i,j}\right\}}_{j=1}^{J}}{\min}\\ \;\{\overset{J}{\underset{j=1}{\sum }}||y_{i,j}-\Phi_{i,j}\cdot x_{i,j}|{|}_{2}^{2}+\alpha \cdot \overset{J}{\underset{j=1}{\sum }}||\Psi \cdot x_{i,j}|{|}_{1}\} \end{array}$$

where ***Ψ*** denotes the 2D sparse representation basis, α is a regularization factor, ||·||_2_ denotes ℓ_2_ norm, and ||·||_1_ denotes ℓ_1_ norm. The model (3) can be solved by some non-linear optimization algorithms, e.g., Alternating Direction Method of Multipliers (ADMM) (Yang et al., 2020), and all reconstructed blocks are spliced into the estimated frame ${\hat{f}}_{i}$. The frame-by-frame model uses only the spatial correlations, so its rate-distortion performance is unsatisfactory. The 3D reconstruction model fully considers the spatial-temporal correlations and it can be represented by

(4) $$\begin{array}{l} {\left\{{\hat{x}}_{i,j}{|}_{i=1}^{L}\right\}}_{j=1}^{J}=arg\underset{{\left\{x_{i,j}{|}_{i=1}^{L}\right\}}_{j=1}^{J}}{\min}\{\overset{L}{\underset{i=1}{\sum }}\overset{J}{\underset{j=1}{\sum }}∥y_{i,j}-\Phi_{i,j}\cdot x_{i,j}{∥}_{2}^{2}\\ \;+\alpha \cdot \overset{L}{\underset{i=1}{\sum }}\overset{J}{\underset{j=1}{\sum }}|\Gamma \cdot \left[x_{1,j},x_{2,j},\cdots \;,x_{L,j}\right]|\} \end{array}$$

where ***Γ*** denotes the 3D sparse representation basis, and it is used to remove the spatial-temporal redundancies between blocks. Though effective, model (4) results in a heavy computational burden. Different from the 3D reconstruction, the distributed reconstruction uses the motion-compensation based prediction technique to expose the spatial-temporal redundancies between blocks. Figure 1 shows the mechanism of MH prediction, which is commonly used in distributed reconstruction. MH prediction collects the spatial-temporal neighboring blocks in key frames to construct an MH matrix ***H***_*i,j*_. According to the motion vector ***v***_*i*_ of ***x***_*i,j*_, the motion-aligned windows ***W***_1_ and ***W***_2_ of sizes *W* × *W* are, respectively, located on the previous and the next key frames, and all candidate blocks in ***W***_1_ and ***W***_2_ are extracted as the hypotheses ${\left\{h_{t}\right\}}_{t=1}^{T}$ of ***x***_*i,j*_, producing ***H***_*i,j*_ = [***h***_1_, ***h***_2_, ⋯ , ***h***_*T*_], in which *T* = *W*^2^. By using MH prediction, the distributed reconstruction is modeled as a Least-Squares (LS) problem as follows:

(5) $$\begin{array}{l} {ŵ}_{i,j}=arg\underset{w}{\min}\left\{∥y_{i,j}-\Phi_{i,j}\cdot H_{i,j}\cdot w{∥}_{2}^{2}+\beta \cdot ∥\Theta \cdot w{∥}_{2}^{2}\right\} \end{array}$$

(6) $$\begin{array}{l} {\hat{x}}_{i,j}=H_{i,j}\cdot {ŵ}_{i,j} \end{array}$$

where ***Θ*** is the Tikhonov matrix, and β is a regularization factor. ***Θ*** is a diagonal matrix and constructed by

(7) $$\begin{array}{l} \Theta =\left[\begin{array}{cc} ∥y_{i,j}-\Phi_{i,j}\cdot h_{1}{∥}_{2} & 0\\ \ddots \\ 0 & ∥y_{i,j}-\Phi_{i,j}\cdot h_{T}{∥}_{2} \end{array}\right] \end{array}$$

With this structure, ***Θ*** assigns weights of small magnitude to hypotheses mostly dissimilar from ***x***_*i,j*_. The LS problem can be fast solved by the Conjugate Gradient algorithm (Zhang et al., 2018), which significantly reduces the computational complexity of distributed reconstruction. Due to the full exploitation of spatial-temporal correlations between blocks, the MH prediction enables the distributed reconstruction to provide superior recovery. From the above, in order to realize a light decoder and ensure a good recovery at the same time, distributed reconstruction is a wise way.

### 2.3. Contexts

Compressive Sensing theory indicates that the sparsity *K* of the signal determines its required number *M* of CS measurements by precise recovery. An empirical rule (Becker and Bobin, 2011) is that the precise recovery can be achieved if

(8) $$\begin{array}{l} M\geq 4\cdot K \end{array}$$

In the block-based CS sampling, this rule can be used to avoid the redundancy or insufficiency of CS measurements for blocks, i.e., adapted by the sparsity, each block is allocated to the appropriate number of CS measurements. The sparsity is defined as the number of coefficients with significant magnitude in a representation, and its calculation has not a strict mathematical formula. For images, the sparsity can be revealed by some features, e.g., edge, variance, gradient, etc., and these features are applied into adaptive allocation, leading to the improvement of recovery quality. The simple features only describe the correlations between pixels, but the structures of blocks are not taken into consideration, thus we require some complex features to improve the efficiency of adaptive allocation. In Ref. Romano and Elad (2016), the self-similarity descriptor (Shechtman and Irani, 2007) is used to extract the contexts of blocks, which represents how similar a central block is to its large surrounding windows. Contexts contain the internal structures and external relations among blocks, and it is a potential feature to better reveal the sparsity variation. The following briefly describes how to extract the contexts in an image.

**Figure 2:**
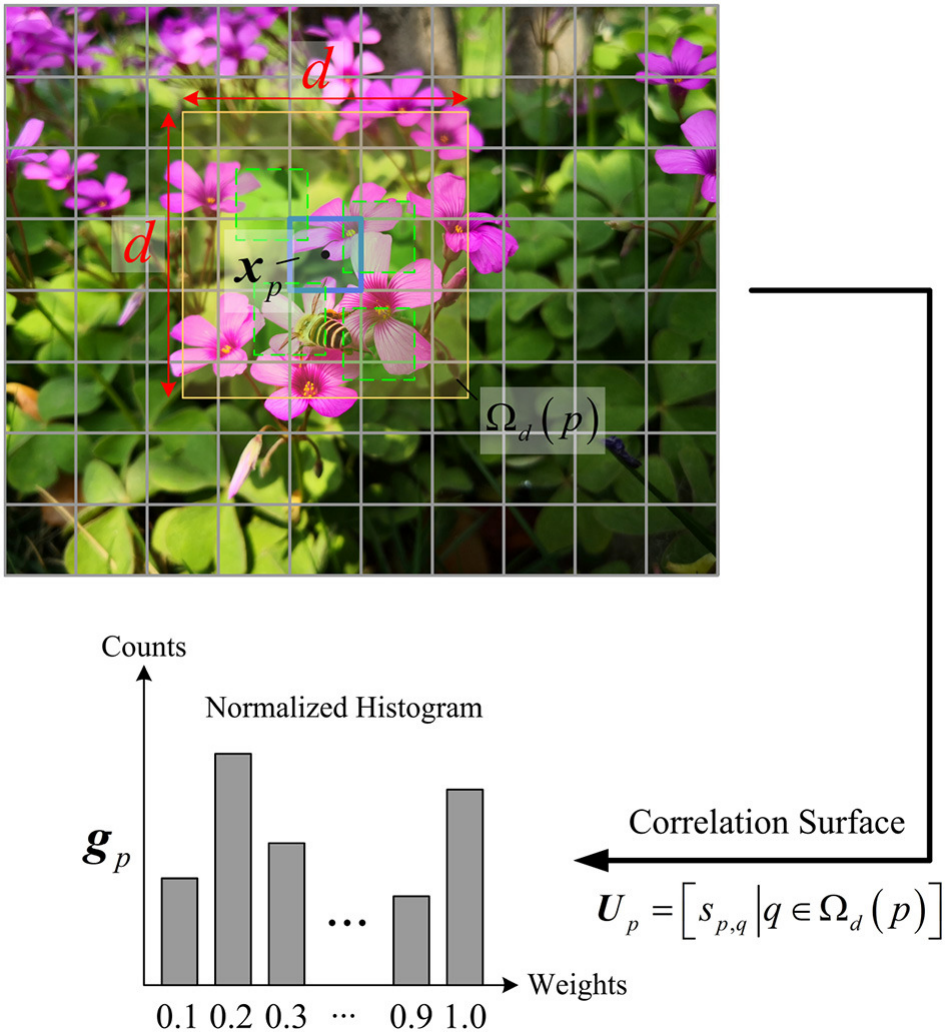
Illustration on the traditional extraction of contexts.

The context feature expresses the similarities between a central block and those of its large surrounding windows. As illustrated in Figure 2, for a central block ***x***_*p*_ in an image, its similarity weights are computed by

(9) $$\begin{array}{l} s_{p,q}=\exp\left\{-\frac{∥x_{p}-x_{q}{∥}_{2}^{2}}{2\sigma^{2}}\right\},\forall q\in \Omega_{d}\left(p\right) \end{array}$$

where ***x***_*q*_ denotes the *q*th surrounding block in a neighborhood Ω_*d*_(*p*) of size *d* × *d*, and σ is a normalization factor. The range of *s*_*p,q*_ is [0, 1], in which a large value indicates that the blocks ***x***_*p*_ and ***x***_*q*_ are highly similar, and a small value indicates that the two are substantially different. All weights constitute a correlation surface ***U***_*p*_ = [*s*_*p,q*_|∀*q* ∈ Ω_*d*_(*p*)], of which the statistics reveal the self-similarity of ***x***_*p*_. To measure the statistics, the correlation surface of ***x***_*p*_ is rearranged into a histogram of *b* bins, of which the normalization is regarded as the context feature ***g***_*p*_ of ***x***_*p*_.

The context feature ***g***_*p*_ is an empirical distribution of the co-occurrences of ***x***_*p*_ in its large surroundings, which measures the correlations between ***x***_*p*_ to its surroundings. When ***g***_*p*_ is biased toward the left bins, it can be concluded that the majority of *s*_*p,q*_ are small, indicating the block ***x***_*p*_ is unique, i.e., it originates from a highly textured and non-repetitive area, so its sparsity is relatively high. When ***g***_*p*_ is biased toward the right bins, it means that most of *s*_*p,q*_ are high, indicating that the block ***x***_*p*_ has many co-occurrences in its surroundings, i.e., it originates from a large flat area, so its sparsity is low. From the above, we can see that the context feature accurately describes the geometric structure of a block with respect to its surrounding blocks, thus it is naturally sensitive to the sparsity variation. However, in CVS, the traditional method is impractical due to the unavailability of original pixels or high computational complexity. Therefore, it is challenging to extract the context feature by using CS measurements of blocks.

## 3. Proposed Context Based CVS System

### 3.1. System Architecture

**Figure 3:**
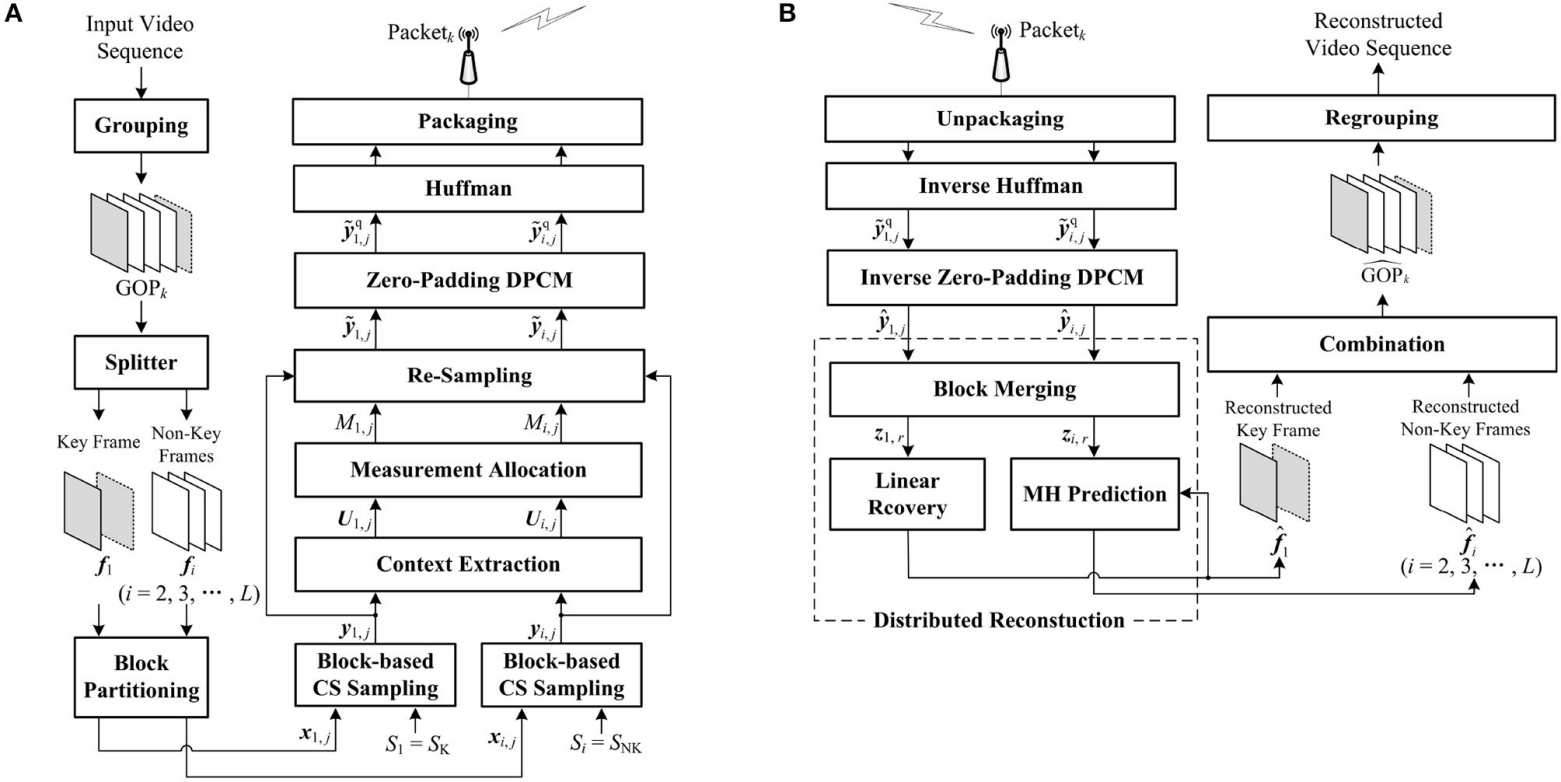
Architecture of the proposed context-based Compressive Video Sensing (CVS) system: **(A)** encoder framework, **(B)** decoder framework.

As shown in Figure 3, we describe the architecture of the proposed context-based CVS system in detail. The input video sequence is divided into several GOPs of length *L*, and each GOP_*k*_ is successively encoded as Packet_*k*_. After receiving this packet, the decoder reconstructs the corresponding ${\hat{\text{GOP}}}_{k}$, and all reconstructed GOPs are regrouped as the entire video sequence.

Figure 3A presents the process of encoding GOP_*k*_. The key frame ***f***_1_ is split from GOP_*k*_, and others ${\left\{f_{i}\right\}}_{i=2}^{L}$ are regarded as the non-key frames. The key frame ***f***_1_ and the *i*th non-key frame ***f***_*i*_ are partitioned into *J* non-overlapping blocks ${\left\{x_{1,j}\right\}}_{j=1}^{J}$ and ${\left\{x_{i,j}\right\}}_{j=1}^{J}$ of size *B* × *B*, respectively. For the key frame ***f***_1_, we set a high subrate *S*_1_ = *S*_K_ to sample the blocks ${\left\{x_{1,j}\right\}}_{j=1}^{J}$ and generate the CS measurements ${\left\{y_{1,j}\right\}}_{j=1}^{J}$ according to Equation (1). The blocks ${\left\{x_{i,j}\right\}}_{j=1}^{J}$ in the non-key frame ***f***_*i*_ are sampled at a low subrate *S*_*i*_ = *S*_NK_, producing the corresponding CS measurements ${\left\{y_{i,j}\right\}}_{j=1}^{J}$ by Equation (1). For ***f***_1_ and ***f***_*i*_, based on the preset subrates, CS measurements are uniformly allocated to each block, however, without considering the structures of blocks, the uniform allocation results in either redundancy or insufficiency of CS measurements for some blocks. To improve the efficiency of block-based CS sampling, the core of the encoder is to perform the adaptive allocation by contexts of blocks. Different from traditional methods, the contexts ***U***_1,*j*_ and ***U***_*i,j*_ of ***x***_1,*j*_ and ***x***_*i,j*_ are, respectively, extracted by using the CS measurements ***y***_1,*j*_ and ***y***_*i,j*_, which makes CVS system more practical. After context extraction, according to the contexts ***U***_1,*j*_ and ***U***_*i,j*_, the numbers of CS measurements of ***x***_1,*j*_ and ***x***_*i,j*_ are modified as *M*_1,*j*_ and *M*_*i,j*_ by adaptive allocation. According to *M*_1,*j*_ and *M*_*i,j*_, by removing the redundancy or supplementing the insufficiency in ***y***_1,*j*_ and ***y***_*i,j*_, ***x***_1,*j*_ and ***x***_*i,j*_ are re-sampled as ${\tilde{y}}_{1,j}$ and ${\tilde{y}}_{i,j}$, respectively. DPCM cannot be used to quantize the adaptive measurements with different numbers. To overcome this defect of DPCM, we fuse zero padding into DPCM and predictively quantize ${\tilde{y}}_{1,j}$ and ${\tilde{y}}_{i,j}$ as ${\tilde{y}}_{1,j}^{\text{q}}$ and ${\tilde{y}}_{i,j}^{\text{q}}$. Finally, all quantized CS measurements are encoded as bits by Huffman and packaged as Packet_*k*_.

Figure 3B presents the process of decoding Packet_*k*_. After unpackaging Packet_*k*_, the inversions of Huffman and zero-padding DPCM are implemented, and the CS measurements of ***x***_1,*j*_ and ***x***_*i,j*_ are recovered as ${\hat{y}}_{1,j}$ and ${\hat{y}}_{i,j}$ which have some quantization errors with their originals ${\tilde{y}}_{1,j}$ and ${\tilde{y}}_{i,j}$. The distributed reconstruction is performed to reconstruct the key frame ***f***_1_ and the non-key frames ${\left\{f_{i}\right\}}_{i=2}^{L}$. To suppress the blocking artifacts in the reconstructed frames, we realize the recovery of large blocks by merging the CS measurements of the spatially neighboring blocks, so the CS measurements of ***f***_1_ and ***f***_*i*_ are updated as ***z***_1,*r*_ and ***z***_*i,r*_ for large blocks. Based on ***z***_1,*r*_, the reconstructed key frame ${\hat{f}}_{1}$ is produced by using a linear recovery model, which rapidly recovers each block by a matrix-vector product. Regarding the previous and the next reconstructed key frames as references, the MH prediction outputs the reconstructed non-key frame ${\hat{f}}_{i}$ by using ***z***_*i,r*_. Finally, all reconstructed frames are combined into ${\hat{\text{GOP}}}_{k}$. Details of the core parts, including contexts extraction, measurements allocation, zero-padding DPCM, and distribution reconstruction, are described in the following subsections.

### 3.2. Context Extraction

**Figure 4:**
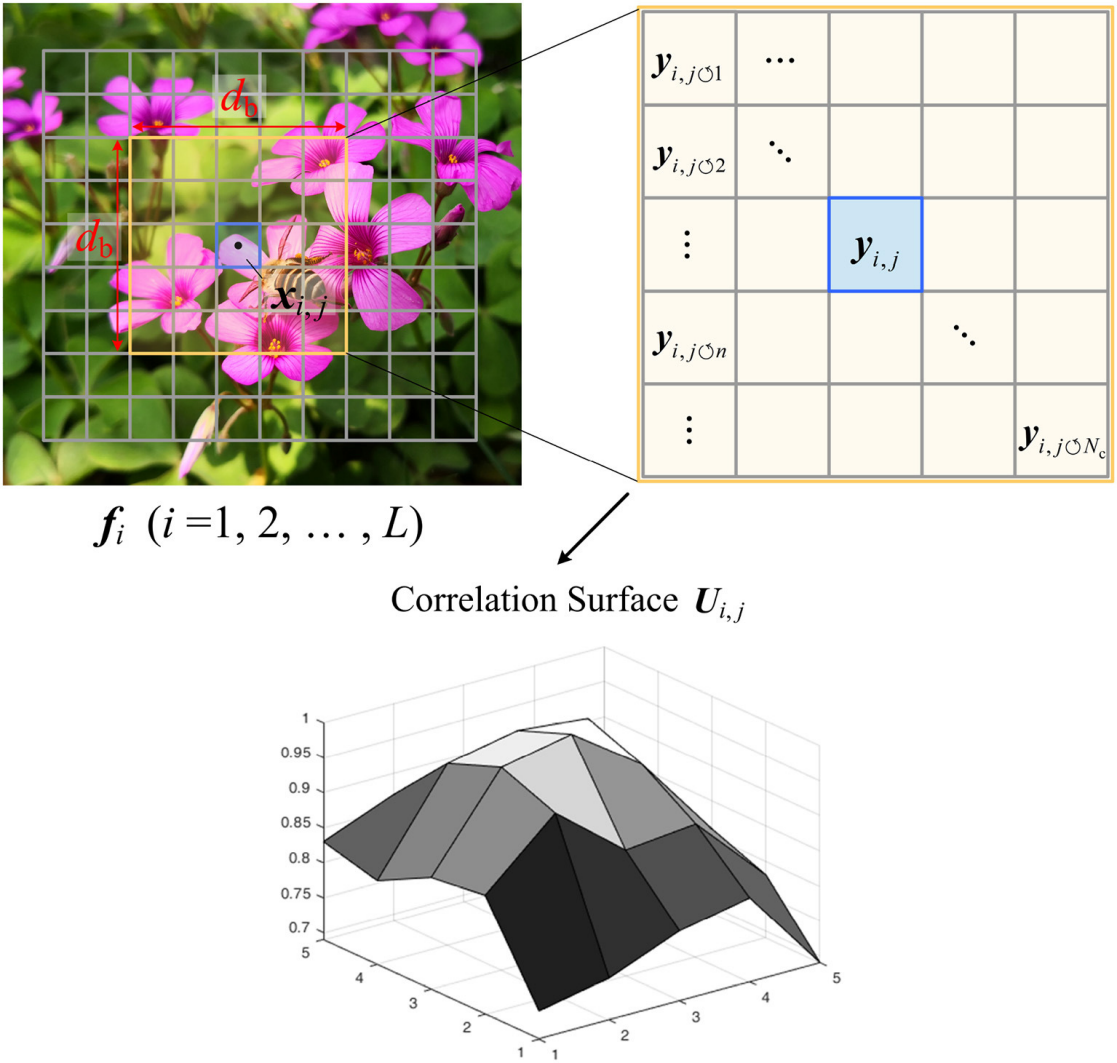
Illustration on contexts extraction based on Compressive Sensing (CS) measurements.

In the proposed CVS system, the context features are extracted by using the CS measurements of blocks. As illustrated in Figure 4, we compute the correlation surface ***U***_*i,j*_ of ***x***_*i,j*_ in ***f***_*i*_ as its contexts, in which *i* = 1, 2, ⋯ , *L*. In the surrounding window of size *d*_b_ × *d*_b_ centered on ***x***_*i,j*_, we cannot extract the original blocks pixel-by-pixel due to the unavailability of original pixels, but can only use the CS measurements ${\left\{y_{i,j↺n}\right\}}_{n=1}^{N_{\text{c}}}$ of non-overlapping blocks ${\left\{x_{i,j↺n}\right\}}_{n=1}^{N_{\text{c}}}$, in which $N_{\text{c}}=d_{\text{b}}^{2}$. According to CS theory, the measurement matrix ***Φ***_*i,j*_ holds the Restricted Isometry Property (RIP) (Candès and Wakin, 2008) for blocks ${\left\{x_{i,j}\right\}}_{j=1}^{J}$, which implies that all pairwise distances between original blocks can be well preserved in the measurement space, i.e.,


(10) $$\begin{array}{l} ∥x_{i,j}-x_{i,j↺n}{∥}_{2}\approx ∥\Phi_{i,j}\cdot x_{i,j}-\Phi_{i,j}\cdot x_{i,j↺n}{∥}_{2}\\ =∥y_{i,j}-y_{i,j↺n}{∥}_{2},\forall n\in \left\{1,2,\cdots \;,N_{\text{c}}\right\} \end{array}$$

where it is noted that all blocks share the same measurement matrix ***Φ***_*i,j*_ due to the uniform allocation. Based on Equation (10), the similarity weights between ***x***_*i,j*_ and ***x***_*i, j*↺*n*_ can be estimated by

(11) $$\begin{array}{l} s_{i,j↺n}=\exp\left\{-\frac{∥y_{i,j}-y_{i,j↺n}{∥}_{2}^{2}}{2\sigma^{2}}\right\},\forall n\in \left\{1,2,\cdots \;,N_{\text{c}}\right\} \end{array}$$

All weights constitute the correlation surface ***U***_*i,j*_ as follows:

(12) $$\begin{array}{l} U_{i,j}=\left[s_{i,j↺n}|\forall n\in \left\{1,2,\cdots \;,N_{\text{c}}\right\}\right] \end{array}$$

To compactly represent the contexts of ***x***_*i,j*_, we compute the mean *u*_*i,j*_ of ***U***_*i,j*_ as the context feature, i.e.,


(13) $$\begin{array}{l} u_{i,j}=\frac{1}{N_{\text{c}}}\overset{N_{\text{c}}}{\underset{n=1}{\sum }}s_{i,j↺n} \end{array}$$

### 3.3. Measurement Allocation

By exploiting the context feature *u*_*i,j*_ of ***x***_*i,j*_, we set the appropriate number of CS measurements for ***x***_*i,j*_, and remove the redundancy or supplement the insufficiency in ***y***_*i,j*_. The magnitudes of context features are high in smooth regions, and the magnitudes are low in the edge and texture regions, so it is found that the experience that the context feature is inversely proportional to the sparsity. Based on this experience, we can describe the distribution on the sparsity degrees of blocks by

(14) $$\begin{array}{l} P_{i,j}=\frac{u_{i,j}^{-1}}{\sum_{j=1}^{J}u_{i,j}^{-1}} \end{array}$$

According to the present subrate *S*_*i*_ of ***f***_*i*_, we construct the allocation model of CS measurements for blocks as follows:

(15) $$\begin{array}{l} M_{i,j}=arg\;\underset{m_{i,j}}{\min}\overset{J}{\underset{j=1}{\sum }}\left(m_{i,j}-P_{i,j}\cdot S_{i}\cdot N\right)\\ \text{s}.\text{t}.\;\overset{j}{\underset{j=1}{\sum }}m_{i,j}=S_{i}\cdot N,m_{i,j}\leq 0.9\cdot N_{\text{b}},m_{i,j}\in {\mathbb{N}}^{+} \end{array}$$

where *N* is the total number of pixels in ***f***_*i*_, *N*_b_ is the block length, *m*_*i,j*_ is a positive integer, and its upper bound is set to be 0.9·*N*_b_. The model (15) is solved according to Algorithm 1 and outputs the final number *M*_*i,j*_ of CS measurements for ***x***_*i,j*_.

**Algorithm 1:** Allocating the appropriate numbers of Compressive Sensing (CS) measurements to blocks.

**Require:** *S*_*i*_ - Subrate of ***f***_*i*_, *P*_*i,j*_ - Distribution on the sparsity of blocks ***x***_*i,j*_, *j* = 1, 2, ⋯ , *J*, *N*- Total number of pixels in ***f***_*i*_, *N*_b_ - Block length;
1: Initial measurement number $m_{i,j}^{\left(0\right)}=\text{Round}\left(P_{i,j}S_{i,j}N\right)$, and Round(·) is a rounding operator;
2: Restrict $m_{i,j}^{\left(0\right)}$ to not be larger than 0.9*N*_b_, i.e., $m_{i,j}^{\left(0\right)}=\text{Min}\left(m_{i,j}^{\left(0\right)},0.9N_{\text{b}}\right)$, in which Min(·) is a minimization operator;
3: Set $M_{\text{sup}}=S_{i}\cdot N-\overset{J}{\underset{j=1}{\sum }}m_{i,j}^{\left(0\right)}$, and *iter* = 0;
4: **while** *M*_sup_ > 0, increment *iter* by 1 **do**
5: **if** *M*_sup_ < *J* **then**
6: Randomly select *M*_sup_ blocks, and their measurement numbers are incremented by 1;
7: Update $m_{i,j}^{\left(iter\right)}$, and set $M_{i,j}=m_{i,j}^{\left(iter\right)}$;
8: **Break**;
9: **else**
10: $m_{i,j}^{\left(iter+1\right)}=m_{i,j}^{\left(iter\right)}+1$
11: *M*_sup_ = *M*_sup_ − *J*
12: **end if**
13: **end while**
14: **return** *M*_*i,j*_, *j* = 1, 2, ⋯ , *J*.

### 3.4. Zero-Padding DPCM

**Figure 5:**
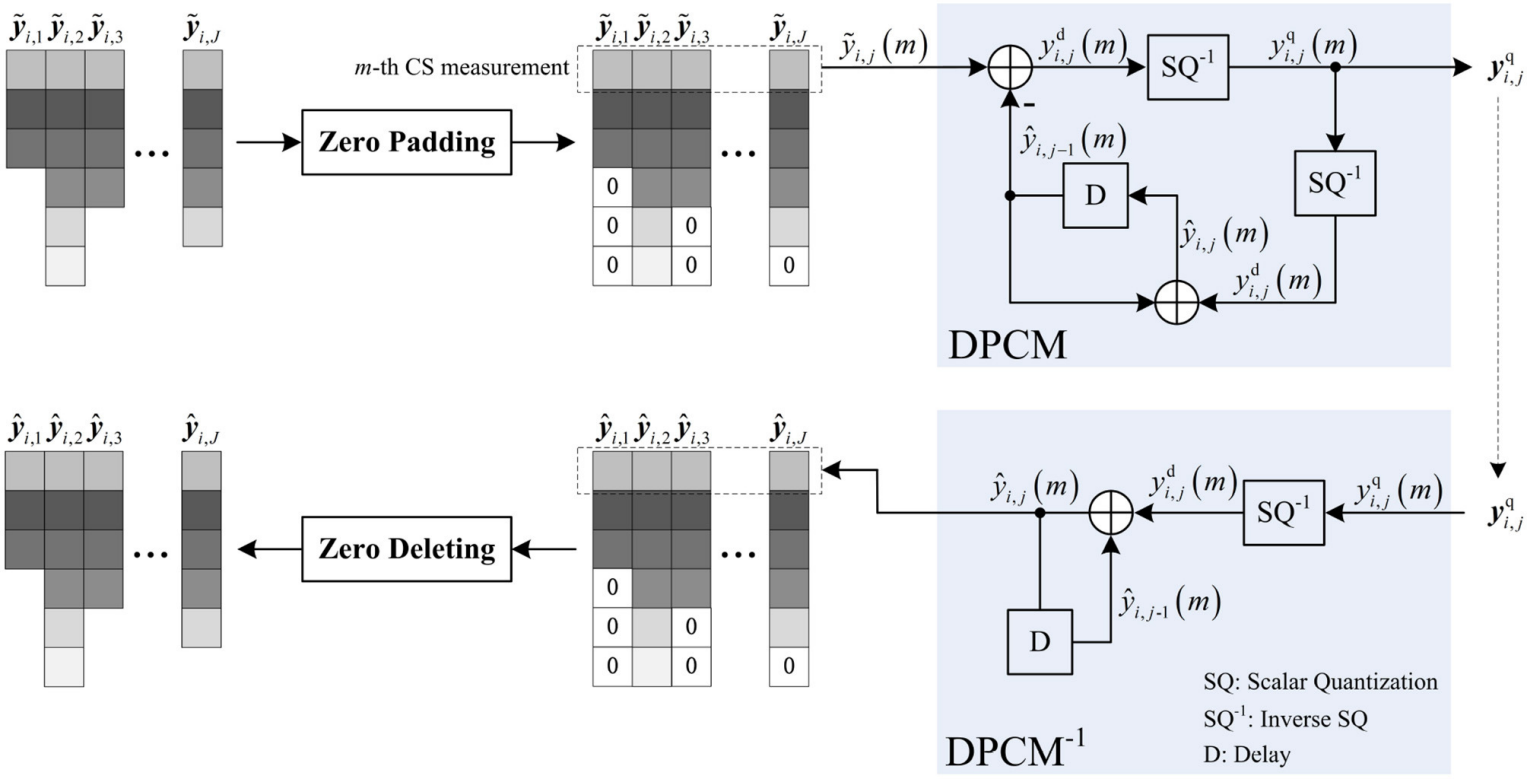
Illustration on zero-padding Differential Pulse Code Modulation (DPCM).

Due to the adaptive allocation, the lengths of the re-sampled CS measurements ${\left\{{\tilde{y}}_{i,j}\right\}}_{j=1}^{L}$ vary. Compared with SQ, DPCM provides better rate-distortion performance by adding the predictive scheme into the quantization of block-based CS measurements. However, DPCM requires that all blocks have the same number of CS measurements, as a result, DPCM cannot be used to quantize ${\left\{{\tilde{y}}_{i,j}\right\}}_{j=1}^{L}$. To make DPCM adapt to the adaptive allocation, we propose zero-padding DPCM, whose implementation is shown in Figure 5. Before inputting ${\tilde{y}}_{i,j}$ to DCPM, we fill zeros in the last of ${\tilde{y}}_{i,j}$ to make its length the same as others. After obtaining the de-quantized CS measurements ${\hat{y}}_{i,j}$, we delete the zeros in the last of ${\hat{y}}_{i,j}$ to recover its original length *M*_*i,j*_. By zero padding, each measurement in ${\hat{y}}_{i,j-1}$ can be used to predict the corresponding measurement in ${\hat{y}}_{i,j}$, and especially when there is predictive measurement ŷ_*i, j*−1_(*m*) of the *m*-th measurement ỹ_*i,j*_(*m*), the residual $y_{i,j}^{\text{d}}\left(m\right)$ can be significantly reduced due to the intrinsic spatial correlation between ${\tilde{y}}_{i,j}$ and ${\tilde{y}}_{i,j-1}$. The rate-distortion curves of the reconstructed *Foreman, Mobile*, and *Football* sequences are presented when zero-padding DPCM and SQ are, respectively, used to quantize the adaptive CS measurements (shown in Supplementary Figure 1), in which the rate-distortion curve is measured in terms of the Peak Signal-to-Noise Ratio (PSNR) in dB and bitrate in bits per pixel (bpp), and the linear recovery algorithm presented in subsection 3.5 is used to recover each video frame. It can be seen that zero-padding DPCM presents competitive performance with SQ at low bitrates but as the bitrate increases, its improvement of performance over SQ is increasingly significant. From these results, we find that the efficiency of zero-padding DPCM relies on the correlation between block-based CS measurements. With insufficient measurements, the correlation is weakened by the filling of excessive zeros, causing the performance degradation, but when measurements are sufficient, a high correlation is maintained, so the performance improvement stands out. From the above, zero-padding DPCM is more suitable for adaptive measurements compared with SQ.

### 3.5. Distributed Reconstruction

**Figure 6:**
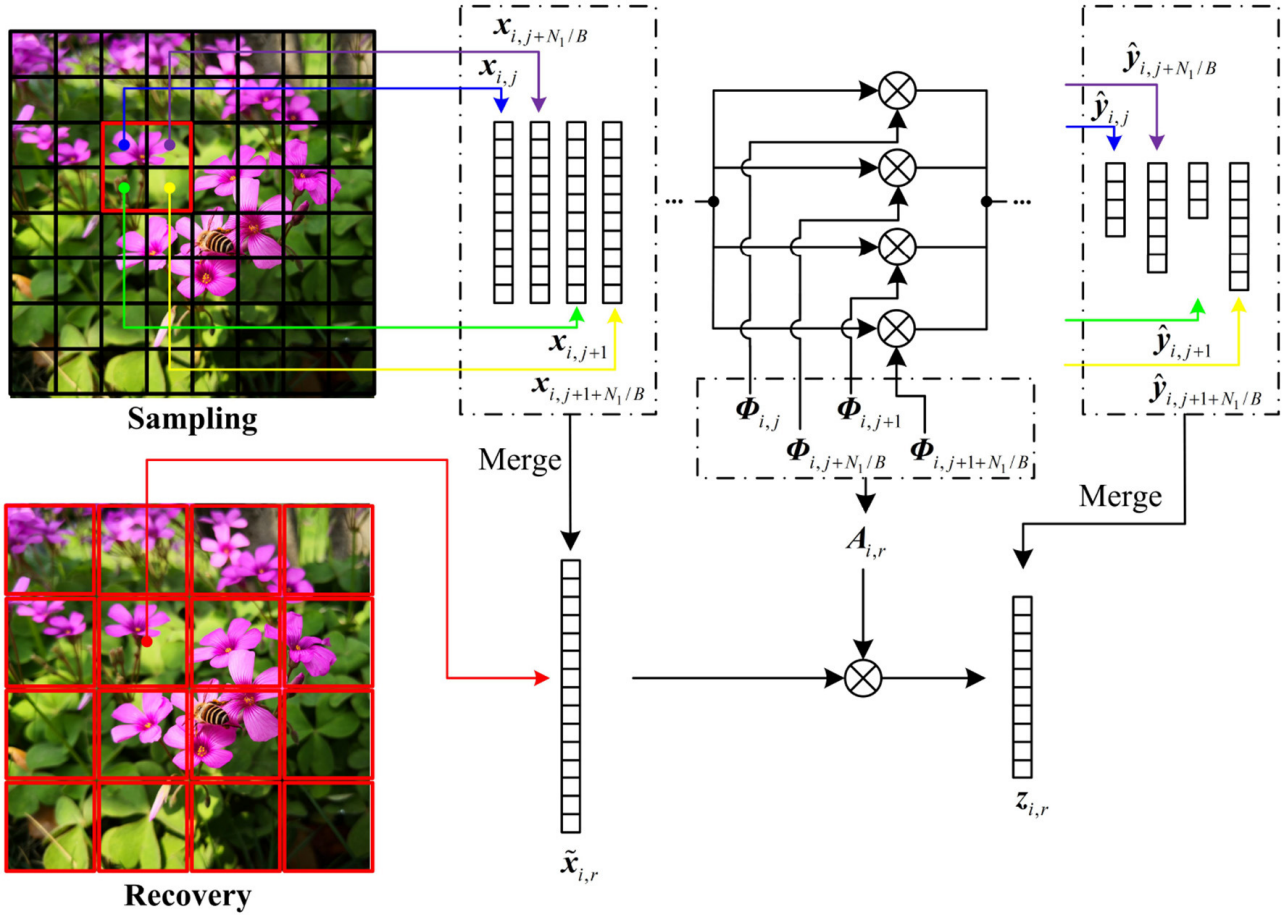
Illustration on block merging when *lev* is set to be 1.

At decoder, the distributed strategy is performed to reconstruct the key frame ***f***_1_ and the non-key frames ${\left\{f_{i}\right\}}_{i=2}^{L}$, in which ***f***_1_ is estimated by a linear recovery model, and ***f***_*i*_ is produced by MH prediction. To highlight the complex structures by contexts, a small block size is more desired at the encoder. However, the small block size causes serious blocking artifacts due to the differences of neighboring blocks in recovery quality. To suppress the blocking artifacts, we merge the CS measurements ${\left\{{\hat{y}}_{i,j}\right\}}_{j=1}^{J}$ of the small blocks ${\left\{x_{i,j}\right\}}_{j=1}^{J}$ into those ${\left\{z_{i,r}\right\}}_{r=1}^{R}$ of the large blocks ${\left\{{\tilde{x}}_{i,r}\right\}}_{r=1}^{R}$ and realize the sampling of small blocks and the recovery of large blocks. The size *B*_*lev*_ × *B*_*lev*_ of large block is set to be

(16) $$\begin{array}{l} B_{lev}=2^{lev}\cdot B,lev=1,2,\cdots \end{array}$$

in which *lev* is a positive integer. The number *R* of large blocks is $N/B_{lev}^{2}$, and it is smaller than the number *J* of small blocks. Figure 6 illustrates the block merging when *lev* is set to be 1. The four neighboring blocks ***x***_*i,j*_, ***x***_*i,j*+1_, ***x***_*i,j*+*N*_1_/*B*_, ***x***_*i,j*+1+*N*_1_/*B*_ are merged into a large block ${\tilde{x}}_{i,r}$, and their CS measurements ${\hat{y}}_{i,j}$, ${\hat{y}}_{i,j+1}$, ${\hat{y}}_{i,j+N_{1}/B}$, and ${\hat{y}}_{i,j+1+N_{1}/B}$ are spliced into ***z***_*i,r*_ in rows, i.e.,

(17) $$\begin{array}{l} z_{i,r}=\left[\begin{array}{c} {\hat{y}}_{i,j}\\ {\hat{y}}_{i,j+1}\\ {\hat{y}}_{i,j+N_{1}/B}\\ {\hat{y}}_{i,j+1+N_{1}/B} \end{array}\right]\approx \Lambda_{i,r}\cdot \left[\begin{array}{c} x_{i,j}\\ x_{i,j+1}\\ x_{i,j+N_{1}/B}\\ x_{i,j+1+N_{1}/B} \end{array}\right] \end{array}$$

(18) $$\begin{array}{l} \Lambda_{i,r}=\left[\begin{array}{cccc} \Phi_{i,j} & \; & \; & 0\\ \; & \Phi_{i,j+1} & \; & \;\\ \; & \; & \Phi_{i,j+N_{1}/B} & \;\\ 0 & \; & \; & \Phi_{i,j+1+N_{1}/B} \end{array}\right] \end{array}$$

in which ***Λ***_*i,r*_ is the diagonal matrix composed of the block measurement matrices ***Φ***_*i,j*_, ***Φ***_*i,j*+1_, ***Φ***_*i,j*+*N*_1_/*B*_, and ***Φ***_*i, j*+1+*N*_1_/*B*_, *N*_1_ is the total number of rows in ***f***_*i*_, and *B* is the block size of the small block. To make ***z***_*i,r*_, the CS measurements of ${\tilde{x}}_{i,r}$, we transform ${\tilde{x}}_{i,r}$ as

(19) $$\begin{array}{l} \left[\begin{array}{c} x_{i,j}\\ x_{i,j+1}\\ x_{i,j+N_{1}/B}\\ x_{i,j+1+N_{1}/B} \end{array}\right]=I\cdot {\tilde{x}}_{i,r} \end{array}$$

in which ***I*** is an elementary column transformation matrix. Plugging Equation (19) into Equation (17), we build the bridge between ${\tilde{x}}_{i,r}$ and ***z***_*i,r*_ by

(20) $$\begin{array}{l} z_{i,r}\approx \Lambda_{i,r}\cdot I\cdot {\tilde{x}}_{i,r}=A_{i,r}\cdot {\tilde{x}}_{i,r} \end{array}$$

in which ***A***_*i,r*_ = ***Λ***_*i,r*_·***I***. According to Equation (20), the large block ${\tilde{x}}_{i,r}$ can be recovered by using ***z***_*i,r*_. When *lev* is set to be larger than 1, the block merging can be done in manner similar to the above.

After the block merging, we use ${\left\{z_{1,r}\right\}}_{r=1}^{R}$ to recover the key frame ***f***_1_. The block ${\tilde{x}}_{1,r}$ of ***f***_1_ is linearly estimated by

(21) $$\begin{array}{l} {\hat{x}}_{1,r}=P_{1,r}\cdot z_{1,r} \end{array}$$

in which ***P***_1,*r*_ is the transformation matrix produced by the following model:

(22) $$\begin{array}{l} P_{1,r}=arg\;\underset{P}{\min}\left\{E\left[∥{\tilde{x}}_{1,r}-P\cdot z_{1,r}{∥}_{2}^{2}\right]\right\} \end{array}$$

in which *E*[·] denotes the expectation function. The model (22) outputs the optimal transformation matrix to minimize the mean square error between ${\tilde{x}}_{1,r}$ and its estimator ${\hat{x}}_{1,r}$, and it can be solved by making the gradient of objective function equal to 0, producing

(23) $$\begin{array}{l} P_{1,r}=E\left[{\tilde{x}}_{1,r}z_{1,r}^{\text{T}}\right]E^{-1}\left[z_{1,r}z_{1,r}^{\text{T}}\right] \end{array}$$

Plugging Equation (20) into Equation (23), we get

(24) $$\begin{array}{l} P_{1,r}={Cor}_{\text{xx}}\cdot A_{1,r}^{\text{T}}{\left(A_{1,r}\cdot {Cor}_{\text{xx}}\cdot A_{1,r}^{\text{T}}\right)}^{-1} \end{array}$$

(25) $$\begin{array}{l} {Cor}_{\text{xx}}=E\left[{\tilde{x}}_{1,r}{\tilde{x}}_{1,r}^{\text{T}}\right] \end{array}$$

in which ***Cor***_xx_ is the auto-correlation matrix of ${\tilde{x}}_{1,r}$, and its element *Cor*_xx_[*m, n*] is estimated as follows:

(26) $$\begin{array}{l} Cor_{\text{xx}}\left[m,n\right]=0.95^{\delta_{m,n}} \end{array}$$

in which δ_*m,n*_ is the Euclidean distance between two pixels ${\tilde{x}}_{1,r}\left(m\right)$ and ${\tilde{x}}_{1,r}\left(n\right)$ in ${\tilde{x}}_{1,r}$. When the subrate is set to be large, the linear recovery model can provide excellent visual quality while costing fewer computations.

## 4. Experimental Results

We evaluate the proposed CVS system on video sequences with various resolutions, including seven CIF (352 × 288) sequences *Akiyo, Bus, Container, Coastguard, Football, Foreman, Hall*, one WQVGA (416 × 240) sequence *BlowingBubbles*, and one 1080p (1920 × 1080) sequence *ParkScene*. In the proposed CVS system, the window size *d*_b_ × *d*_b_ and the normalization factor σ are, respectively, set to be 11 × 11 and 10 for the context extraction, the window size *W* × *W* and the regularization factor β are, respectively, set to be 21 × 21 and 0.25 for the MH prediction, and the measurement matrix is produced by Gaussian distribution. First, we discuss the effects of different block sizes on the proposed CVS system. Second, we evaluate the performance improvement resulting from the used context extraction. Finally, we compare the proposed CVS system with two state-of-the-art CVS systems: SS-CVS (Li et al., 2020) and MH-RTIK (Chen C. et al., 2020) in terms of the rate-distortion performance. PSNR is used to evaluate the qualities of reconstructed video sequences, and the bitrate denotes the average amount of bits per pixel to encode a video sequence. The variation of PSNR with bitrate is called the rate-distortion performance. The computational complexity is measured by the execution time. Experiments are implemented with MATLAB on a workstation with 3.30-GHz CPU and 8 GB RAM.

### 4.1. Effects of Block Sizes

In the proposed CVS system, in order to highlight the complex structures by contexts, we desire a small block size at encoder, but at decoder, a large block size is desired to suppress the blocking artifacts in the reconstructed video frames. We set a block-size pair (*B, B*_*lev*_), in which *B* and *B*_*lev*_ are the block sizes for sampling and recovery, respectively, and evaluate the effects of different block-size pairs on the reconstruction qualities of key frames and non-key frames.

**Figure 7:**
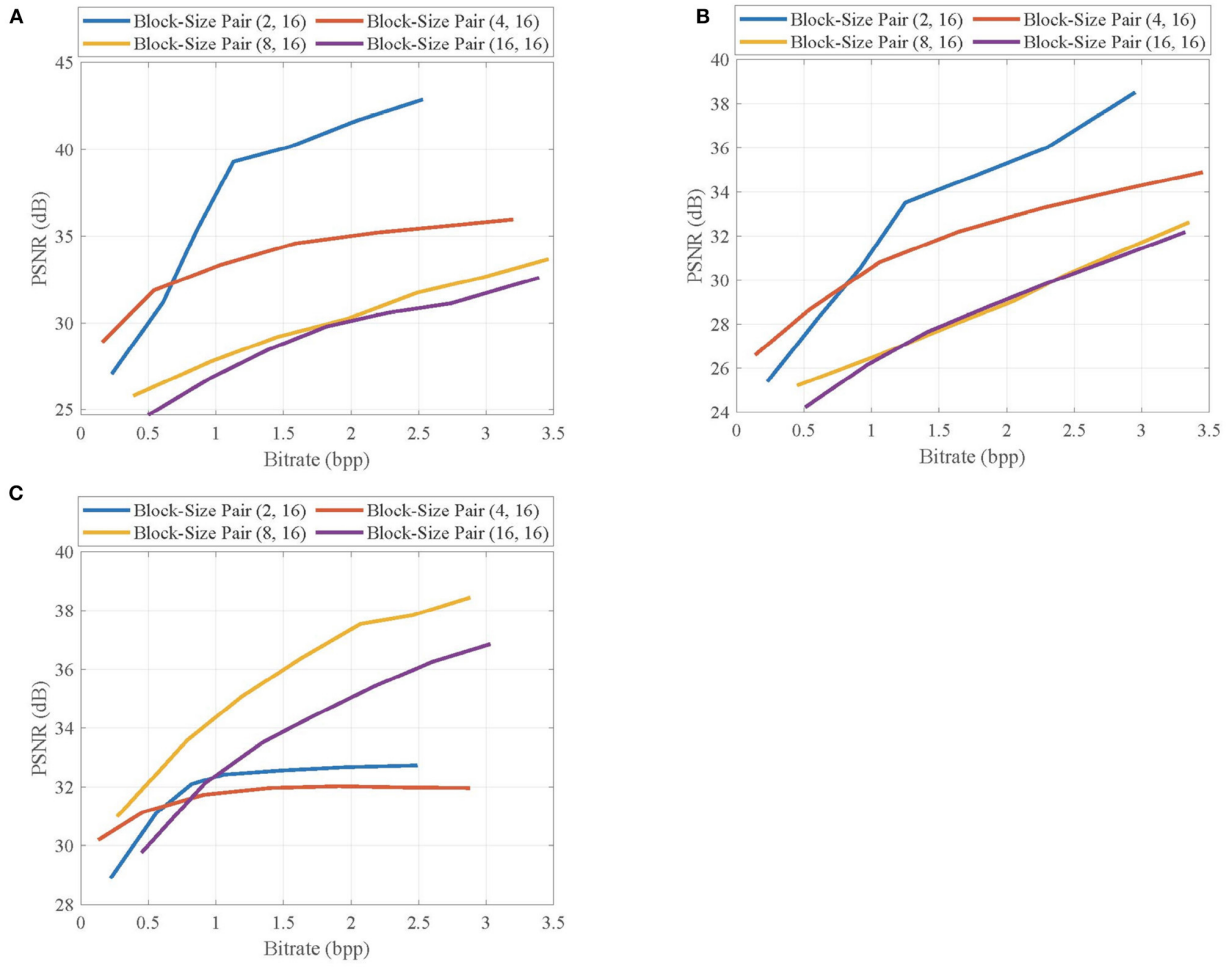
Rate-distortion curves of the reconstructed key frames in **(A)** *Foreman*, **(B)** *BlowingBubbles*, and **(C)** *ParkScene* sequences at different block-size pairs.

First, we select the first frames of *Foreman, BlowingBubbles*, and *ParkScene* sequences as the key frames, which are linearly recovered, and show their rate-distortion curves at different block-size pairs in Figure 7. For *Foreman* and *BlowingBubbles* with the low resolution, the block-size pair (4, 16) achieves higher PSNR values than others with low bitrates, but the rate-distortion curve for the block-size pair (2, 16) rapidly increases as the bitrate increases and significant PSNR gains are achieved when compared with other block-size pairs. These results indicate that the small blocks used in adaptive allocation and large blocks for linear recovery fit together well. For *ParkScene* with high resolution, when the block size *B* for sampling is set to be too small, e.g., *B* = 2, no block can contain sufficient structures, causing the rate-distortion performance to degenerate as the bitrate increases, but a suitable block size for sampling is set, e.g., *B* = 8, PSNR gains can be significantly improved.

**Figure 8:**
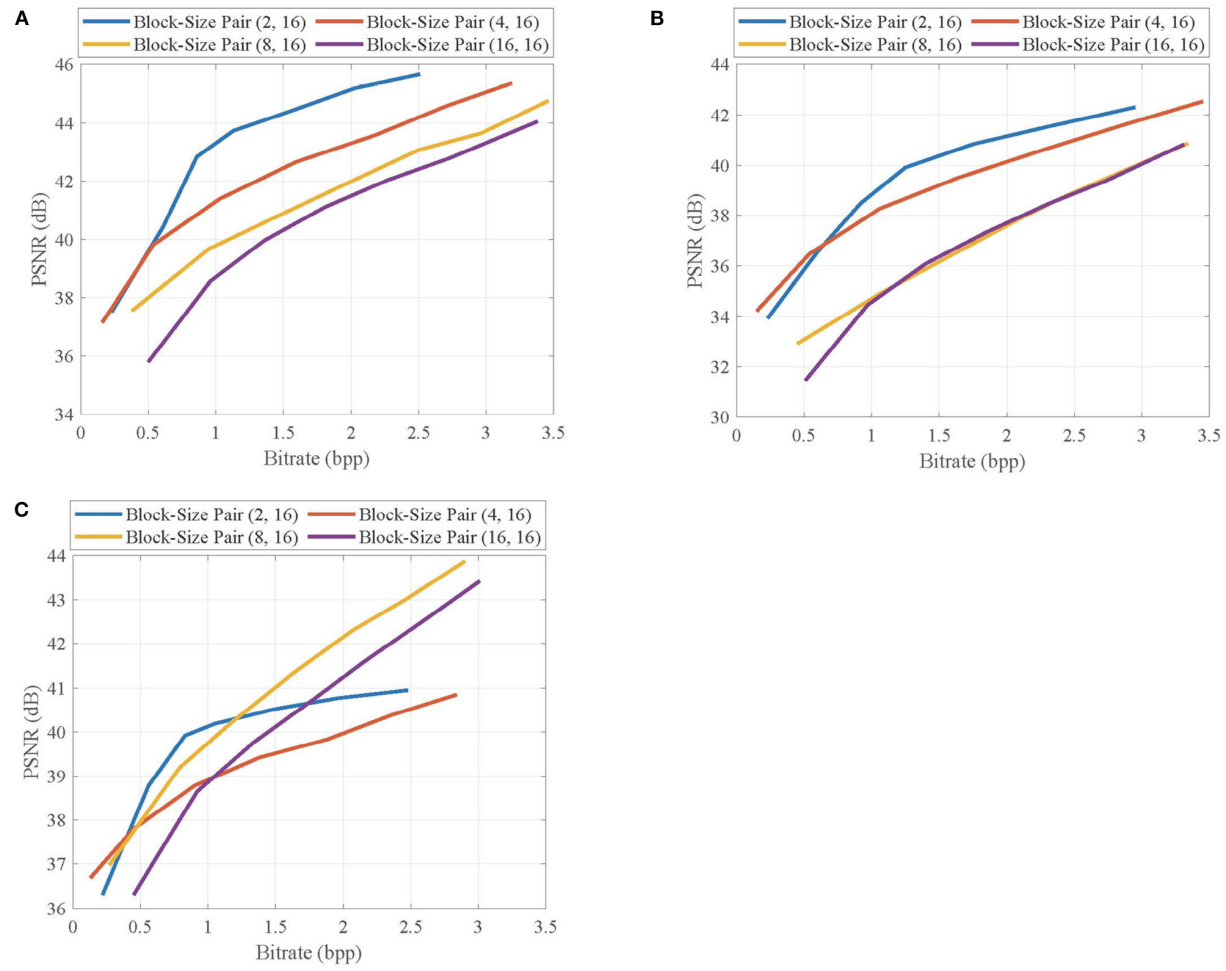
Rate-distortion curves of the reconstructed non-key frames in **(A)** *Foreman*, **(B)** *BlowingBubbles*, and **(C)** *ParkScene* sequences at different block-size pairs.

Then, we select the second frames of *Foreman, BlowingBubbles*, and *ParkScene* sequences as the non-key frames, which are recovered by MH prediction based on the reconstructed previous and next key frames at the subrate 0.7, and show their rate-distortion curves at different block-size pairs in Figure 8. Similar to the results from key frames, for *Foreman* and *BlowingBubbles*, the better rate-distortion performance is achieved when the block-size pair is set to be (2, 16), and for *ParkScene*, in order to prevent the loss of structures, the block size for sampling is appropriately set to be 8.

Given the above, we can see that the bad effects resulting from the extraction of contexts can be suppressed by the block merging, therefore, the quality improvement from contexts-based allocation is further enhanced.

### 4.2. Effects of Contexts

In the proposed CVS system, the contexts are extracted from CS measurements and used to adaptively allocate the CS measurements for blocks, leading to the improvement of reconstruction quality. To verify the validity of contexts from CS measurements on the quality improvement, we evaluate the effects of different allocation schemes on the rate-distortion performance of the proposed CVS system. The uniform allocation is used as a benchmark, and the adaptive allocation uses the contexts extracted from CS measurements and original pixels, respectively.

**Figure 9:**
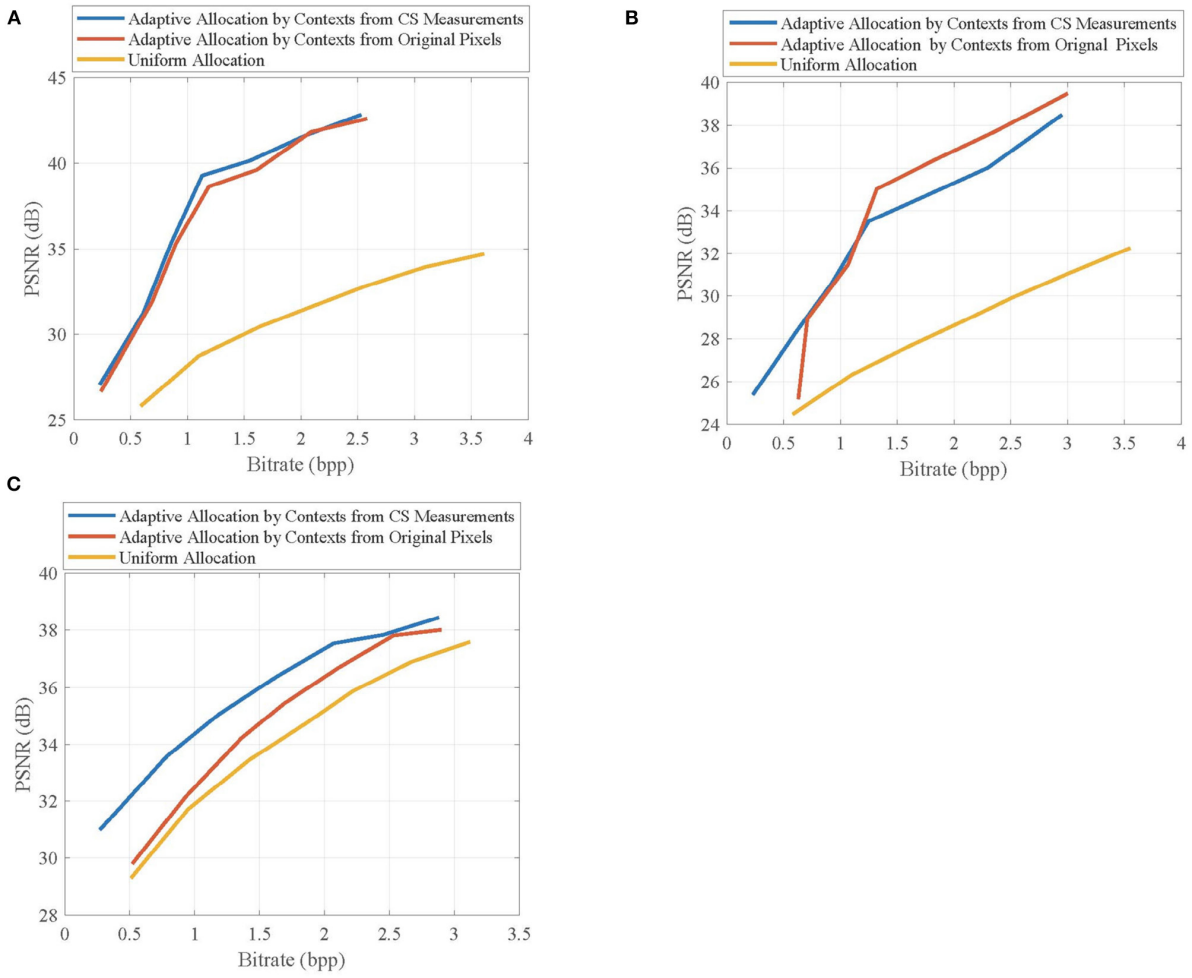
Rate-distortion curves of the reconstructed key frames in **(A)** *Foreman*, **(B)** *BlowingBubbles*, and **(C)** *ParkScene* sequences when using different allocation schemes. For *Foreman* and *BlowingBubbles*, the block-size pair is set to be (2, 16), and for *ParkScene*, the block-size is set to be (8, 16).

Figure 9 shows the rate-distortion curves of the reconstructed key frames when using different allocation schemes, in which the key frames are, respectively, taken from the first frames of *Foreman, BlowingBubbles*, and *ParkScene* sequences. It can be seen that adaptive allocation outperforms uniform allocation in PSNR values at any bitrate, indicating that contexts contribute to quality improvement. Importantly, the contexts from CS measurements are competitive with those from original pixels, and their performance gaps are very small, which means that CS measurements can better represent the contexts of blocks.

**Figure 10:**
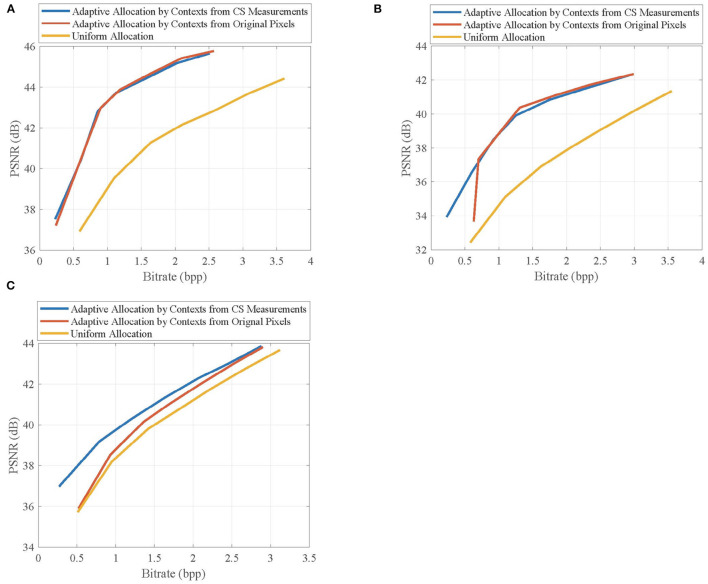
Rate-distortion curves of the reconstructed non-key frames in **(A)** *Foreman*, **(B)** *BlowingBubbles*, and **(C)** *ParkScene* sequences when using different allocation schemes. For *Foreman* and *BlowingBubbles*, the block-size pair is set to be (2, 16), and for *ParkScene*, the block-size is set to be (8, 16).

Figure 10 shows the rate-distortion curves of the reconstructed non-key frames when using different allocation schemes, in which the non-key frames are, respectively, taken from the second frames of *Foreman, BlowingBubbles*, and *ParkScene* sequences. It can be seen that the adaptive allocation is still effective for MH prediction, and it can significantly improve the rate-distortion performances when compared with uniform allocation. The contexts from CS measurements have similar efficiency of allocation to that of contexts from original pixels, which proves that the merits of adaptive allocation can still be maintained in the measurement domain.

The above results indicate that the contexts extracted by CS measurements prompt the adaptive allocation to improve the reconstruction quality of CVS system, which makes the proposed CVS system more suitable to the applications with limited resources.

### 4.3. Performance Comparisons

We evaluate the performance of the proposed CVS system by comparing it with the two state-of-the-art CVS systems: SS-CVS (Li et al., 2020) and MH-RTIK (Chen C. et al., 2020). To make a fair comparison, we keep the parameter settings of SS-CVS and MH-RTIK in their original reports, some important details are repeated as follows:

1. SS-CVS: the system consists of one base layer and one enhancement layer; the block size is set to be 16; the length of GOP is 10; the subrate of key frame is set to be 0.9; the dimension of the subspace is 10; the number of subspaces is 50.
2. MH-RTIK: the sub-block extraction is used; the number of hypotheses is 40; the block size is set to be 16; the length of GOP is 2; the subrate of key frame is set to be 0.7.

In addition, we employ SQ and Huffman in SS-CVS and MH-RTIK to compress the CS measurements. For the proposed CVS system, the block-size pair is set to be (2, 16) for CIF and QWVGA sequences and (8, 16) for 1080P sequences, the subrate *S*_K_ of key frame is set to be 0.7, the results under the GOP length *L* = 2 are compared with those of MH-RTIK, and the results under the GOP length *L* = 10 are compared with those of SS-CVS.

**Figure 11:**
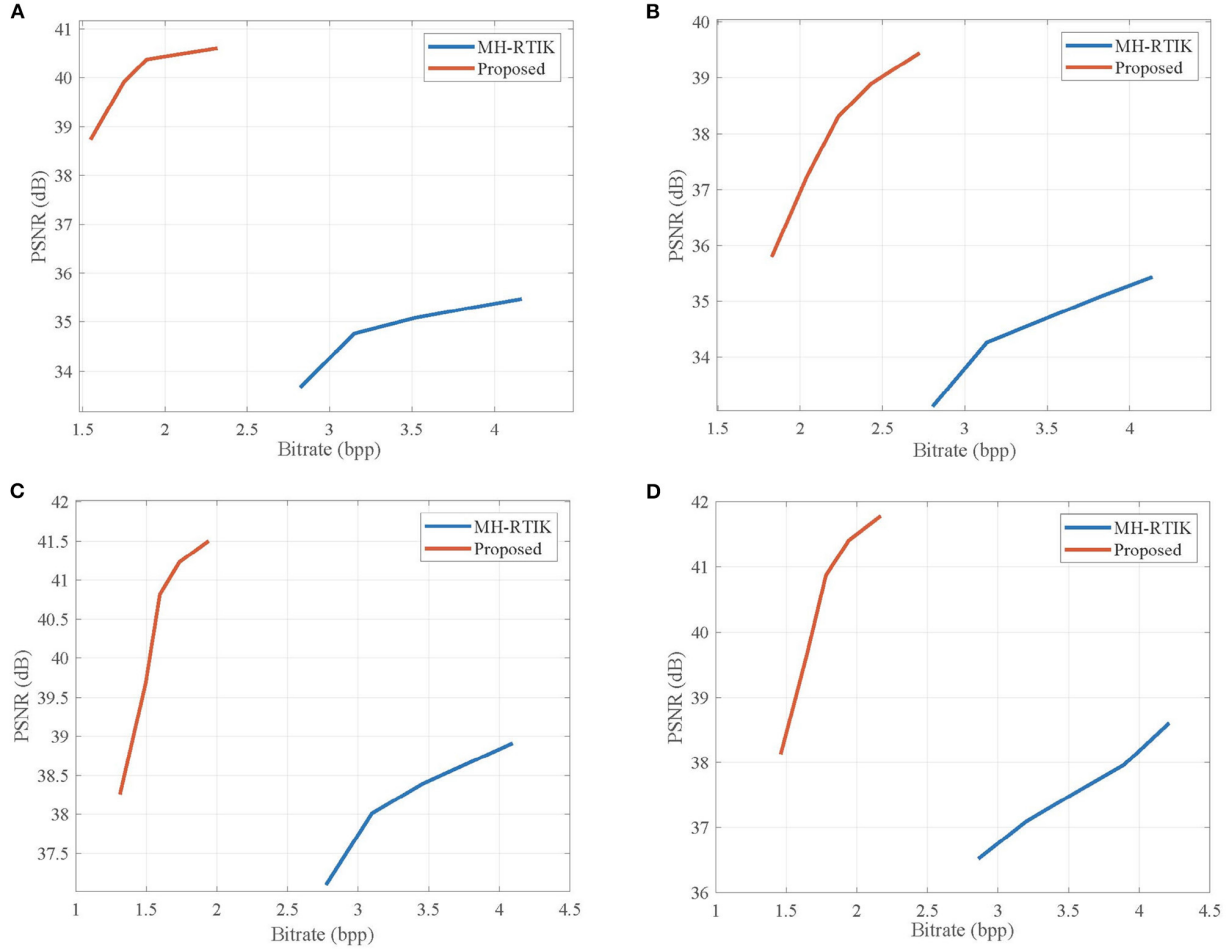
Rate-distortion curves obtained by the proposed CVS system and Multi-Hypothesis TIKhonov (MH-TIK) (Chen C. et al., 2020) for **(A)** *Container*, **(B)** *Coastguard*, **(C)** *Hall*, and **(D)** *Foreman* sequences. Note that the length *L* of GOP is set to be 2.

**Figure 12:**
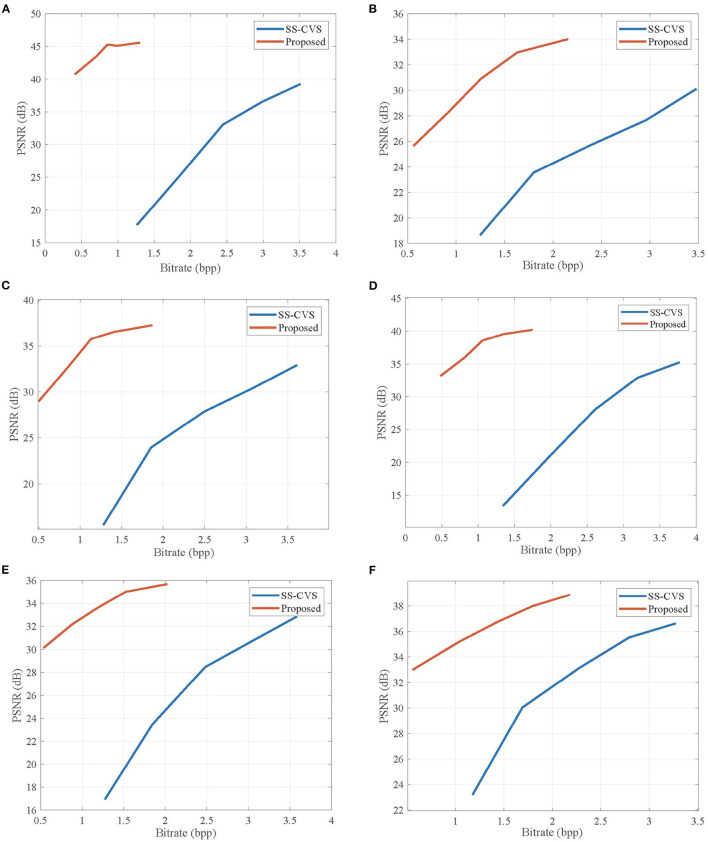
Rate-distortion curves obtained by the proposed CVS system and Scalable Structured CVS (SS-CVS) (Li et al., 2020) for **(A)** *Akiyo*, **(B)** *Bus*, **(C)** *Football*, **(D)** *Foreman*, **(E)** *BlowingBubble*, and **(F)** *ParkScene* sequences. Note that the length *L* of GOP is set to be 10.

Table 1 lists the average PSNR values for the reconstructed video sequences by the proposed CVS system, SS-CVS, and MH-RTIK when the subrate *S*_NK_ of non-key frame varies from 0.1 to 0.5. Compared with MH-RTIK, the proposed CVS system achieves obvious PSNR gains at any subrate, e.g., the average PSNR gain is 2.824 dB for the *Foreman* sequence. Compared with SS-CVS, the proposed CVS system also presents higher PSNR values at any subrate, and especially for low subrates, PSNR gains are significant, e.g., when the subrate is 0.1, PSNR gains are 9.82, 13.20, and 19.78 dB for *ParkScene, BlowingBubble, Foreman* sequences, respectively. Figures 11, 12 show the rate-distortion curves for the proposed CVS system, MH-RTIK, and SS-CVS. Due to the implementation of zero-padding DPCM, the performance improvement of the proposed CVS system is further enhanced when compared with MH-RTIK and SS-CVS. By the objective evaluation of the reconstruction quality, it can be indicated that the proposed CVS system can significantly improve the qualities of the reconstructed video sequences.

**Table 1:** Average Peak Signal-to-Noise Ratio (PSNR) (dB) for reconstructed video sequences by the proposed Compressive Video Sensing (CVS) system, Scalable Structured CVS (SS-CVS) (Trevisi et al., 2020), and Multi-Hypothesis Reweighted TIKhonov (MH-RTIK) (Chen C. et al., 2020) at subrates 0.1 to 0.5.

**Sequence**	**Resolution**	**Algorithm**	**Subrate** ***S***_**NK**_				
			**0.1**	**0.2**	**0.3**	**0.4**	**0.5**
**GOP Length** ***L*** **= 2**							
*Container*	CIF	MH-RTIK	33.67	34.76	35.08	35.28	35.47
		Proposed	**38.74**	**39.92**	**40.38**	**40.47**	**40.61**
*Coastguard*		MH-RTIK	33.12	34.26	34.69	35.08	35.43
		Proposed	**35.80**	**37.22**	**38.30**	**38.89**	**39.45**
*Hall*		MH-RTIK	37.10	38.01	38.39	38.65	38.91
		Proposed	**38.26**	**39.69**	**40.82**	**41.23**	**41.50**
*Foreman*		MH-RTIK	36.52	37.09	37.56	37.96	38.60
		Proposed	**38.13**	**39.66**	**40.87**	**41.41**	**41.78**
**GOP Length** ***L*** **= 10**							
*Akiyo*	CIF	SS-CVS	17.70	24.80	33.06	36.55	39.23
		Proposed	**40.75**	**43.50**	**45.28**	**45.09**	**45.56**
*Bus*		SS-CVS	18.65	23.57	25.71	27.67	30.10
		Proposed	**25.65**	**38.31**	**30.97**	**32.97**	**34.00**
*Football*		SS-CVS	15.52	23.95	27.87	30.33	32.93
		Proposed	**28.98**	**32.67**	**35.78**	**36.55**	**37.28**
*Foreman*		SS-CVS	13.40	20.51	28.07	32.90	35.25
		Proposed	**33.18**	**36.00**	**38.55**	**39.54**	**40.22**
*BlowingBubble*	QWVGA	SS-CVS	16.93	23.50	28.47	30.70	32.84
		Proposed	**30.13**	**32.17**	**33.58**	**35.01**	**35.68**
*ParkScene*	1080P	SS-CVS	23.19	30.04	33.14	35.53	36.62
		Proposed	**33.01**	**35.18**	**36.79**	**37.97**	**38.67**

Table 2 lists the average encoding time (s/frame) and decoding time (s/frame) on video sequences with different resolutions for the proposed CVS system, SS-CVS, and MH-RTIK. We compute the average execution time on the range [0.1, 0.5] of subrate *S*_NK_ for the proposed CVS system and compare it with that of MH-RTIK for CIF sequences. The encoding speed of the proposed CVS system is slowed down due to the contexts-based adaptive allocation, and its encoding time is 0.63 s per frame, larger than that of MH-RTIK. Assisted by the simple linear recovery, the proposed CVS system reduces the decoding complexity, and only costs 4.48 s to reconstruct a video frame, however, MH-RTIK requires 19.34 s per frame. Under the subrate *S*_NK_ = 0.6, the execution time of the proposed CVS algorithm is compared with that of SS-CVS for the CIF, QWVGA, and 1080P video sequences, respectively. Compared with SS-CVS, the proposed CVS system costs less encoding time, and the encoding time does not dramatically increase as the resolution increases, e.g., for 1080P sequence, the proposed CVS system only costs 1.83 s per frame, but SS-CVS costs 108.10 s. In SS-CVS, the subspace clustering and the basis derivation are implemented at the encoder, and they lead to more encoding costs than the adaptive allocation in the proposed CVS system. The proposed CVS system costs less decoding time than SS-CVS, and its decoding costs also grow more slowly when compared with SS-CVS, e.g., for 1080P sequence, the proposed CVS system costs 162.47 s per frame, and the SS-CVS costs 401.8 s. The heavy computational burdens for SS-CVS derive from the non-linear subspace learning, but the decoding complexity of the proposed CVS system is limited benefiting from the linear recovery and prediction. From the above, we can see that the proposed CVS system still keeps a low computational complexity while providing better rate-distortion performance.

**Table 2:** Average encoding time (s/frame) and decoding time (s/frame) on video sequences with different resolutions for the proposed CVS system, SS-CVS (Li et al., 2020), and MH-RTIK (Chen C. et al., 2020).

**Resolution**	**Algorithm**	**Encoding Time (s/frame)**	**Decoding Time (s/frame)**
**Average on Subrates** ***S***_**NK**_ **0.1 to 0.5**			
CIF	MH-RTIK	0.17	19.34
	Proposed	0.63	4.48
**Average on Subrates *S*_NK_ = 0.6**			
CIF	SS-CVS	5.40	21.22
	Proposed	0.64	7.79
QWVGA	SS-CVS	4.90	17.23
	Proposed	0.64	7.69
1080P	SS-CVS	108.10	401.8
	Proposed	1.83	162.47

## 5. Conclusion

In this article, a context-based CVS system is proposed to improve the visual quality of the reconstructed video sequences. At the encoder, the CS measurements are adaptively allocated for blocks according to the contexts of video frames. Innovatively, the contexts are extracted by CS measurements. Although the extraction of contexts is independent of original pixels, these contexts can still better reveal the structural complexity of each block. To guarantee better rate-distortion performance, the zero-padding DPCM is proposed to quantize these adaptive measurements. At the decoder, the key frames are reconstructed by linear recovery, and these non-key frames are reconstructed by MH prediction. Thanks to the effectiveness of context-based adaptive allocation, the simple recovery schemes also provide the comfortable visual quality. Experimental results show that the proposed CVS system improves the rate-distortion performances when compared with two state-of-the-art CVS systems, including MH-RTIK and SS-CVS, and guarantees a low computational complexity.

As the research in this article is exploratory, there are many intriguing questions that future work should consider. First, the estimation of block sparsity should be analyzed in mathematics. Second, we will investigate how to fuse the quantization into adaptive allocation. More importantly, we will deploy the adaptive CVS system on an actual hardware platform.

## Data Availability Statement

The original contributions presented in the study are included in the article/Supplementary Material, further inquiries can be directed to the corresponding author/s.

## Author Contributions

RL: designed the study and drafted the manuscript. YY: conducted experiments and analyzed the data. FS: critically reviewed and improved the manuscript. All authors have read and approved the final version of the manuscript.

## Funding

This work was supported in part by the Project of Science and Technology, Department of Henan Province in China (212102210106), National Natural Science Foundation of China (31872704), Innovation Team Support Plan of University Science and Technology of Henan Province in China (19IRTSTHN014), and Guangxi Key Laboratory of Wireless Wideband Communication and Signal Processing of China.

## Conflict of Interest

The authors declare that the research was conducted in the absence of any commercial or financial relationships that could be construed as a potential conflict of interest.

## Publisher's Note

All claims expressed in this article are solely those of the authors and do not necessarily represent those of their affiliated organizations, or those of the publisher, the editors and the reviewers. Any product that may be evaluated in this article, or claim that may be made by its manufacturer, is not guaranteed or endorsed by the publisher.
