# Supplementary material for: Green Visual Sensor of Plant: An Energy-Efficient Compressive Video Sensing in the Internet of Things
Source: Front Plant Sci. 2022 Feb 28;13:849606. doi: 10.3389/fpls.2022.849606 (PMC8918948; doi:10.3389/fpls.2022.849606)
Supplement: Supplementary file 1 [file Data_Sheet_1.pdf]

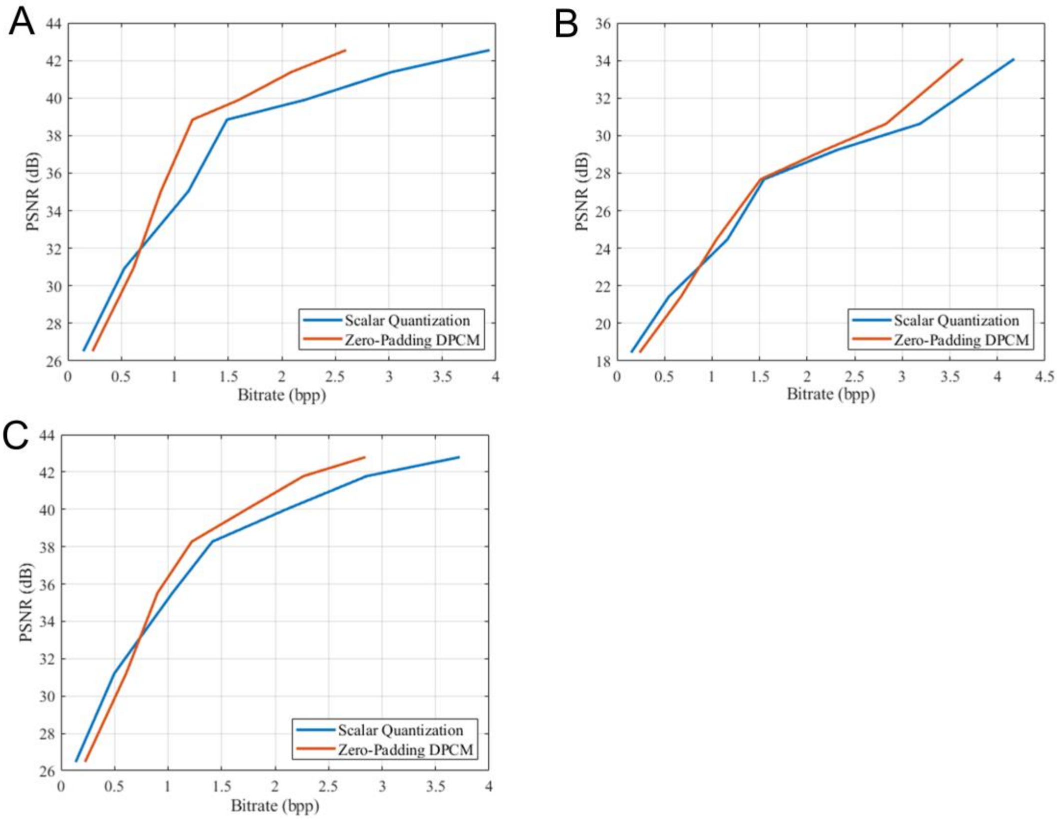

Figure S1. Rate-distortion curves of the reconstructed (A) *Foreman*, (B) *Mobile* and (C) *Football* sequences when the zero-padding DPCM and SQ are respectively used to quantize the adaptive CS measurements.
